# Occult Macular Dysfunction Syndrome: Identification of Multiple Pathologies in a Clinical Spectrum of Macular Dysfunction with Normal Fundus in East Asian Patients: EAOMD Report No. 5

**DOI:** 10.3390/genes14101869

**Published:** 2023-09-26

**Authors:** Yu Fujinami-Yokokawa, Lizhu Yang, Kwangsic Joo, Kazushige Tsunoda, Xiao Liu, Mineo Kondo, Seong Joon Ahn, Hui Li, Kyu Hyung Park, Hisateru Tachimori, Hiroaki Miyata, Se Joon Woo, Ruifang Sui, Kaoru Fujinami

**Affiliations:** 1 Department of Health Policy and Management, Keio University School of Medicine, Tokyo 160-8582, Japan; y.fujinami@keio.jp (Y.F.-Y.);; 2 Laboratory of Visual Physiology, Division of Vision Research, National Institute of Sensory Organs, NHO Tokyo Medical Center, Tokyo 152-8902, Japan; 3 UCL Institute of Ophthalmology, London EC1V 9EL, UK; 4 Division of Public Health, Yokokawa Clinic, Suita 564-0083, Japan; 5 Department of Ophthalmology, Peking Union Medical College Hospital, Peking Union Medical College and Chinese Academy of Medical Sciences, Beijing 100193, China; 6 Department of Ophthalmology, Seoul National University Bundang Hospital, Seoul National University College of Medicine, Seongnam 13620, Republic of Korea; 7 Division of Vision Research, National Institute of Sensory Organs, NHO Tokyo Medical Center, Tokyo 152-8902, Japan; 8 Southwest Hospital, Army Medical University, Chongqing 400715, China; 9 Key Lab of Visual Damage and Regeneration & Restoration of Chongqing, Chongqing 400715, China; 10 Department of Ophthalmology, Mie University Graduate School of Medicine, Mie 514-8507, Japan; 11 Department of Ophthalmology, Hanyang University Hospital, Hanyang University College of Medicine, Seoul 04763, Republic of Korea; 12 Department of Ophthalmology, Seoul National University Hospital, Seoul National University College of Medicine, Seoul 03080, Republic of Korea; 13 Endowed Course for Health System Innovation, Keio University School of Medicine, Tokyo 160-8582, Japan; 14 Moorfields Eye Hospital, London EC1V 2PD, UK

**Keywords:** occult macular dystrophy, miyake disease, *RP1L1*, *CRX*, *GUCY2D*, non-*RP1L1*

## Abstract

Occult macular dystrophy (OMD) is the most prevalent form of macular dystrophy in East Asia. Beyond *RP1L1*, causative genes and mechanisms remain largely uncharacterised. This study aimed to delineate the clinical and genetic characteristics of OMD syndrome (OMDS). Patients clinically diagnosed with OMDS in Japan, South Korea, and China were enrolled. The inclusion criteria were as follows: (1) macular dysfunction and (2) normal fundus appearance. Comprehensive clinical evaluation and genetic assessment were performed to identify the disease-causing variants. Clinical parameters were compared among the genotype groups. Seventy-two patients with OMDS from fifty families were included. The causative genes were *RP1L1* in forty-seven patients from thirty families (30/50, 60.0%), *CRX* in two patients from one family (1/50, 2.0%), *GUCY2D* in two patients from two families (2/50, 4.0%), and no genes were identified in twenty-one patients from seventeen families (17/50, 34.0%). Different severities were observed in terms of disease onset and the prognosis of visual acuity reduction. This multicentre large cohort study furthers our understanding of the phenotypic and genotypic spectra of patients with macular dystrophy and normal fundus. Evidently, OMDS encompasses multiple Mendelian retinal disorders, each representing unique pathologies that dictate their respective severity and prognostic patterns.

## 1. Introduction

Occult macular dystrophy (OMD; OMIM:613587), first described by Miyake et al. in 1989 [1,2,3,4,5], is the most prevalent form of macular dystrophy in the East Asian population [6,7,8,9,10,11,12,13,14,15,16]. This non-syndromic autosomal dominant (AD) disorder is characterised by progressive loss of visual acuity (VA) in both eyes despite the essentially normal fundus appearance and normal full-field electroretinogram (ffERG) [1,2,3]. Thus, functional assessment to detect confined macular dysfunction using focal macular ERG, multifocal ERG (mfERG), or pattern ERG is key to making a diagnosis [1,2,9,17,18,19,20,21,22,23].

The advancement of detailed morphological assessments enables the observation of characteristic features on spectral-domain optical coherence tomography (SD-OCT) images [6,9,11,24,25,26,27,28,29,30,31,32]. A ‘classical’ photoreceptor microstructure phenotype showing blurring of the ellipsoid zone (EZ) and the absence of the interdigitation zone (IZ) is frequently observed at the fovea in typical patients with AD-OMD, and subtle morphological changes predominantly affecting IZ and other EZ and IZ patterns at the parafovea have been found in patients with mild OMD [6,28].

Monoallelic sequence variants of the retinitis pigmentosa 1 like 1 (*RP1L1*; OMIM: 608581) gene were first identified as being responsible for OMD in two AD families in 2010 [25,33]. The immunohistochemistry assessment in monkeys demonstrated the expression of the *RP1L1* protein in rod/cone photoreceptor cells, suggesting its role in morphological and functional maintenance [33]. Consequently, many *RP1L1* variants have been reported in families with AD-OMD [6,10,11,14,15,19,34,35]. Since the identifying of biallelic sequence variants illustrating the loss of function in patients with retinitis pigmentosa (RP) in 2013, *RP1L1* variants in an autosomal recessive (AR) manner have also been reported [12,19,30,36,37,38].

Various phenotypic features have been reported in OMD caused by monoallelic *RP1L1* variants (*RP1L1*-OMD; Miyake disease) and other disorders with macular dysfunction and normal fundus (non-*RP1L1* OMD), and the disease spectrum of occult macular dysfunction syndrome (OMDS), including hereditary and possibly non-hereditary diseases, was first suggested in 2016 [11]. Recently, patients with inherited macular dystrophy and normal fundus caused by genes other than *RP1L1* have been reported (hereditary non-*RP1L1* OMD) [39,40]. Therefore, OMDS has been assumed to include multiple underlying pathologies that affect the macula without an abnormal fundus appearance.

The East Asia Inherited Retinal Disease Society (EAIRDs; https://www.eairds.org/, accessed on 1 May 2023.) was established in 2016 to investigate and treat IRD in the East Asian population [6]. The first report described the detailed characteristics of East Asian patients with *RP1L1*-OMD and revealed a wide range of clinical findings [6]; the second report described the objective functional phenotypes detected using mfERG [7]; and the third report described the scotoma patterns of varying clinical severities [8]. However, the spectrum of OMDS has yet to be comprehensively investigated because of the lack of data resources on non-*RP1L1* OMD. Therefore, in this study, we aimed to thoroughly delineate the clinical and genetic characteristics of OMDS in a large cohort of East Asian patients.

## 2. Materials and Methods

### 2.1. Patients

The research protocol conformed to the principles of the Declaration of Helsinki and was approved by the local ethics committees of the participating institutions in Japan, South Korea, and China (National Hospital Organization Tokyo Medical Centre, National Institute of Sensory Organs [NISO], Bundang Hospital of Seoul National University (SNUBH), Peking Union Medical College Hospital, Peking Union Medical College (PUMCH), Chinese Academy of Medical Sciences) (Ref: R19-030, R 21-108, R22-028, JS-2056, B-1105/127-014). Written informed consent was obtained from all of the participants.

Patients who were clinically diagnosed with OMDS between 1 June 2016 and 1 July 2023 were recruited via the EAIRDs online database. The inclusion criteria were as follows: (1) evidence of macular dysfunction confirmed by electrophysiological assessment and (2) the presence of a normal fundus appearance shown by colour fundus photography or fundoscopy. Patients with any signs of associated non-ocular abnormalities were excluded. The affected family members of the proband who met the diagnostic criteria were also included. Genetic diagnosis was performed in each country, and pathogenicity assessment of the identified variants was centrally conducted at NISO. All cases were re-confirmed for diagnosis by a principal investigator from each country (K.F., S.J.W. and R.S.), and cases where there were differences in opinion between principal investigators were excluded from this study. Some data on the included cases were partially published in previous EAOMD reports [6,7,8].

### 2.2. Clinical Investigation

A comprehensive clinical examination was performed, including the conversion of the best-corrected decimal VA (BCVA) to the logarithmic minimum resolution median angle (logMAR). Detailed disease history, visual symptoms, gender, age, disease onset (when the patient first noted symptoms or was diagnosed), and disease duration (from onset to examination) were recorded. Ophthalmological examinations included funduscopic observation, fundus photographs, fundus autofluorescence images, SD-OCT, visual fields (VF), ffERG, and mfERG, according to the international standard guidelines of the International Society of Clinical Electrophysiology of Vision [41,42,43,44].

FfERGs were recorded with different recording systems in the 3 institutes [6]. A custom-made system with a UTAS BigShot (LKC, MD, USA) and a MEB-9400K Neuropack S1 (NIHON KOHDEN, Tokyo, Japan); and a LE4000 (Tomey, Aichi, Japan) was used in NISO. A UTAS system (LKC, MD, USA) was used in SNUBH. RETIPort system (Roland Consult, Wiesbaden, Germany) was used in PUMCH. The mfERGs were recorded with different recording systems in the 3 institutes [7]. The Visual Evoked Response Imaging System (VERIS Clinic 5.0.9; Electro-Diagnostic Imaging, San Mateo, CA, USA) was used with a 61-hexagon stimulus element (5 eccentric rings) in NISO. VERIS (Clinic 6.0) was used with a 103-hexagon stimulus element (6 rings) in PUMCH. The RETIscan system (Roland Consult, Brandenburg, Germany) with a 61-hexagon stimulus element (5 rings), VERIS (Clinic 4.0) with a 103-hexagon stimulus element (6 rings), and UTAS (3.5.0; LKC Technologies, Gaithersburg, MD, USA) with a 61-hexagon stimulus element (5 rings) were used in SNUBH.

### 2.3. Classification of Clinical Parameters (VF, mfERG, SD-OCT)

Based on the clinical parameters, patients were classified into the following subgroups: VF, mfERG, and SD-OCT findings, according to previous reports [6,7,8]. Patients were classified into two patterns based on VF findings using standard automated visual field testing: pattern 1, central scotoma; and pattern 2, other scotomas (e.g., paracentral scotoma) or no scotoma [8]. Based on the mfERG findings, the patients were classified into three functional groups: group 1, paracentral dysfunction with relatively preserved central/peripheral function; group 2, homogeneous central dysfunction with preserved peripheral function; and group 3, extensive dysfunction across the entire recorded area [7]. ‘Classical’ SD-OCT findings were characterised by the blurring of the EZ and the absence of IZ at the macula according to the previous literature [3,25,33]. The ‘non-classical’ findings show changes not consistent with the classical phenotype, such as a relatively preserved EZ with focal loss of IZ, loss of both EZ and IZ in focal areas, or atrophic changes within the outer retinal layers [6].

### 2.4. Molecular Genetic Analysis

All monoallelic *RP1L1* variants detected in the EAOMD cohort were reviewed and in silico molecular genetic analyses were conducted in accordance with previous publications [6,45]. The pathogenicity of each detected *RP1L1* variant was assessed according to the guidelines of the American College of Medical Genetics and Genomics (ACMG) [46,47].

### 2.5. Genotype Subgroup Classification

Genotypic subgroup classification of the OMD phenotype was performed based on the underlying causative genes *RP1L1*, *CRX*, *GUCY2D*, and cases in which causative genes were not detected (ND).

### 2.6. Comparison of Clinical Parameters and Clinical Classifications (VF/mfERG/SD-OCT)

Clinical parameters, including age, onset, disease duration, BCVA, VF classification, mfERG classification, and SD-OCT classification, were compared between patients with each causative gene group: *RP1L1*, *CRX*, *GUCY2D*, and ND. Kaplan-Meier survival analysis was used to assess BCVA (logMAR 0.22 and logMAR 1.00). For genotype subgroups with a limited number of patients (fewer than three), a literature search was conducted for cases with identical variants to compile a comprehensive clinical dataset for a ‘genotype review plus (R+)’. The percentage of patients in each VF/mfERG/SD-OCT subgroup was calculated for each genotype.

### 2.7. Statistical Analysis

Statistical analyses were performed using Excel Tokei 4.04, provided by Social Research and Information Inc. (Social Survey Research Information Co., Ltd., Tokyo, Japan) and SPSS Statistics (Version 29, Statistical Package for the Social Sciences; IBM Corp, Armonk, NY, USA). A chi-square test was applied to compare the categorical data (clinical classifications).

Statistical significance was set at *p* < 0.05. For genotype R+ analysis, cases previously reported in the literature presenting the identical variants detected in this study were incorporated [16,40,48,49,50,51,52,53,54,55,56,57,58,59]. Kaplan–Meier survival analysis was employed, utilising BCVA metrics (LogMAR 0.22 and LogMAR 1.00 corresponding to the driver’s licence level and social blind level in Japan, respectively), to generate survival curves to predict the natural progression of VA deterioration. The log-rank test was used to compare the survival curves across the different genotype groups.

## 3. Results

### 3.1. Patients

Seventy-two patients from fifty families who were clinically diagnosed with OMDS were included in this study. Detailed information is provided in Table S1.

### 3.2. Demographics and Clinical Findings

The median age of onset in the 72 patients was 37.5 (range, 2–89) years. Fifty-four patients reported reduced vision as the main complaint (54/72, 75.0%). Seven patients (7/72, 9.7%) had no symptoms and five of these asymptomatic individuals were assessed because of a history of AD inheritance in their families, while the other two were identified during routine medical check-ups. Photophobia was reported in conjunction with reduced vision in 20 patients (12/72, 27.8%). The median disease duration in the 72 patients was 10.5 (range, 0–63 years). Fourteen patients were immediately recruited after experiencing visual symptoms or undergoing ocular examinations (duration, 0 years). The median logMAR BCVA of the 72 cases was 0.52 and 0.52 for the right and left eyes, respectively (range, −0.18 to 1.52 for the right and −0.18 to 1.7 for the left).

### 3.3. Classification of Clinical Parameters (VF, mfERG, SD-OCT)

VF, mfERG, and SD-OCT classifications were performed; detailed information is provided in Table S1. The VF data were available for 67 patients. A central scotoma pattern was detected in 50 participants (VF pattern 1, 50/67, 74.6%), and other or no scotoma patterns were found in 17 patients (VF pattern 2, 13/67, 25.3%). The mfERG data were available for fifty-eight patients; nine patients demonstrated paracentral dysfunction with relatively preserved central and peripheral function (mfERG group 1, 9/58, 15.5%), forty-two patients showed homogeneous central dysfunction with preserved peripheral function (mfERG group 2, 42/58, 72.4%), and seven patients had widespread dysfunction in the recorded area (mfERG group 3, 7/34, 8.87%). The SD-OCT classification was available for 72 patients. Classical characteristics of blurring of the EZ and absence of the IZ were demonstrated in 42 patients (classical SD-OCT, 42/72, 58.3%). Non-classical changes that were not consistent with the classical phenotype, such as a relatively preserved EZ with focal loss of the IZ, loss of both the EZ and IZ in focal areas, or atrophic changes within the outer retinal layers, were observed in 30 patients (non-classical SD-OCT, 30/72, 41.7%).

### 3.4. Molecular Genetics

Information on each genotype group is summarised in Table 1. The genetic data of 51 patients from 33 families are summarised in Table 2 and the detailed results of the in silico analyses are presented in Table S2. Some of the genetic information has been published elsewhere and the reference list is presented in Table 2.

The causative genes were *RP1L1* in forty-seven patients from thirty families (30/50, 60.0%; *RP1L1* genotype group), *CRX* in two patients from one family (1/50, 2.0%; *CRX* genotype group), and *GUCY2D* in two patients from two families (2/50, 4.0%; *GUCY2D* genotype group). No disease-causing genes were detected in 21 patients from 17 families (17/50, 34.0%; ND group). The detected variants include nine heterozygous *RP1L1* variants, one heterozygous *CRX* variant, and two heterozygous *GUCY2D* variants; *RP1L1*, NM_178857.6: c.133C>T, p.Arg45Trp; c.3581C>T, p.Thr1194Met/c.3587C>T, p.Thr1196Ile complex; c.3593C>T p.Ser1198Phe; c.3596C>G, p.Ser1199Cys; c.3599G>A, p.Gly1200Asp; c.3599G>C, p.Gly1200Ala; c.3602T>G, p.Val1201Gly; c.3602T>C, p.Val1201Ala; *CRX*, NM_000554.6: c.128G>A, p.Arg43His; *GUCY2D*, NM_000180.4: c.2747T>C, p.Ile916Thr; c.2513G>A, p.Arg838His. Three recurrent variants were identified: *RP1L1*, p.R45W (16/33, 18.2%), p.S1199C (7/33, 21.2%), and *GUCY2D*, p.I916T (2/33, 6.1%).

### 3.5. Demographics for Each Genotype Group

**Figure 1:**
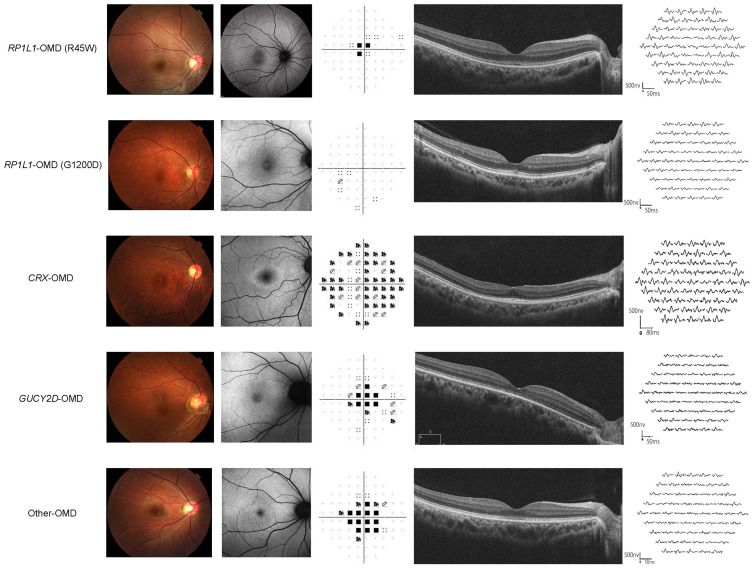
Representative cases with occult macular dysfunction syndrome in each genotype group. The clinical presentation of typical cases in each genotype group (*RP1L1*, *CRX*, *GUCY2D*, and cases in which the causative genes were not detected [ND]); fundus photographs, fundus autofluorescence images, static visual fields (30-2), spectral-domain optical coherence tomography (SD-OCT) images, and multifocal electroretinogram (mfERG). OMD, occult macular dystrophy. Top low (patient 8): 19-year-old female, best-corrected decimal visual acuity (BCVA) to the logarithmic minimum resolution median angle (logMAR) 0.82 in the right eye (RE) and 0.82 in the left eye (LE). Second low (patient 44): 51-year-old female, logMAR BCVA RE −0.08 LE −0.08. Third low (patient 48): 35-year-old male, logMAR BCVA RE 0.22 LE 0.4. Forth low (patient 51): 55-year-old female, logMAR BCVA RE 1.0 LE 1.1. Bottom low (patient 60): 29-year-old male, logMAR RE 0.4 LE 0.4.

**Figure 2:**
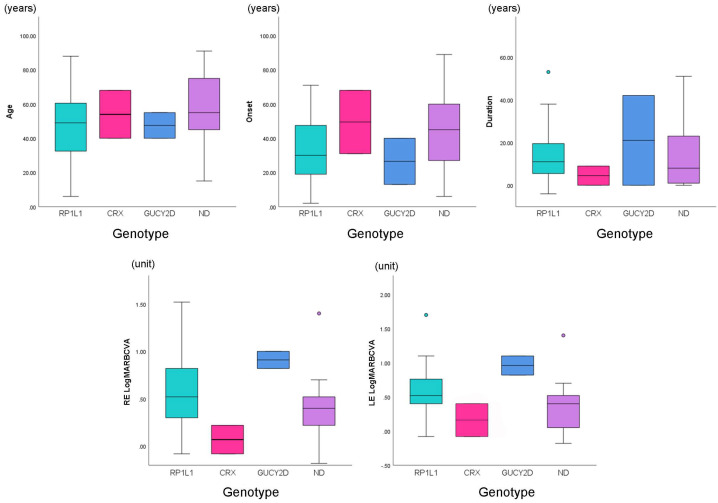
Comparison of age, onset, duration, and best-corrected visual acuity (BCVA) among *RP1L1*, *CRX*, *GUCY2D*, and other genotype (ND) groups. Age, onset, duration, and logarithm of the minimum angle of resolution best-corrected visual acuity (logMAR BCVA) were compared among the *RP1L1*, *CRX*, *GUCY2D*, and ND genotype groups. No statistically significant differences were found in the clinical parameters, although there was a trend toward earlier onset and more severe BCVA in the *RP1L1* and *GUCY2D* genotype groups. ND, not detected.

The demographic data for each genotype are summarised in Table 1 and representative cases are presented in Figure 1. The box plots of the clinical parameters for each genotype group are demonstrated in Figure 2. AD inheritance was detected in twenty-three *RP1L1* families (23/30, 76.7%), one *CRX* family (1/1, 100.0%), and one *GUCY2D* family (1/2, 50.0%). In the ND genotype group, there were five families with AD inheritance (5/17, 29.4%) and two families with AR inheritance (2/17, 11.8%). The median ages for the *RP1L1*, *CRX*, *GUCY2D*, and ND genotype groups were 49.0 (range, 6–88), 54.0 (range, 40–68), 47.5 (range, 40–55), and 55.0 (range, 15–91), respectively. The median age of onset for the *RP1L1*, *CRX*, *GUCY2D*, and ND genotype groups was 30.0 (range, 2–71), 49.5 (range, 31–68), 26.5 (range, 13–40), and 45.0 (range, 6–49), respectively. The median duration of the disease for the *RP1L1*, *CRX*, *GUCY2D*, and ND genotype groups was 11.0 (range, 0–63), 4.5 (range, 0–9), 21.0 (range, 0–42), and 8.0 (range, 0–51), respectively.

### 3.6. Clinical Parameters and Classifications for Each Genotype Group

**Figure 3:**
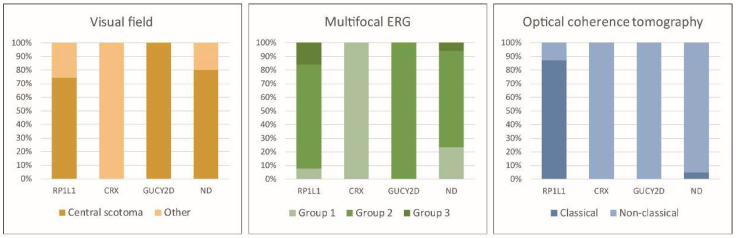
Visual field (VF), multifocal electroretinogram (mfERG), and spectral-domain optical coherence tomography (SD-OCT) classifications for each genotype group. The proportions of VF patterns, mfERG groups, and SD-OCT classifications were compared among the genotype groups. A severe VF pattern with a central scotoma was frequently detected in the *GUCY2D*, ND, and *RP1L1* genotype groups. Classical SD-OCT findings were detected in forty-one patients (41/47, 87.2%) with the *RP1L1* genotype group, while only one case with classical SD-OCT findings was detected in the ND genotype group. mfERG group 2 was frequently found in the *RP1L1*, *GUCY2D*, and ND genotype groups. ND, not detected.

The median logMAR BCVA in the right/left eye for the *RP1L1*, *CRX*, *GUCY2D*, and ND genotype groups were 0.52 (range, −0.08–1.52)/0.52 (range, −0.08–1.70), 0.07 (range, −0.08–0.22)/0.16 (range, −0.08–0.4), 0.91 (range, 0.82–1.00)/0.96 (range, 0.82–1.10), and 0.40 (range, 0.18–1.40)/0.40 (range, 0.18–1.40). VF data were available for forty-three *RP1L1*, two *CRX*, two *GUCY2D*, and 20 ND patients (Figure 3). The number of VF pattern 1/2 detected in the *RP1L1*, *CRX*, *GUCY2D*, and ND genotype groups was 32 (74.4%)/11 (25.6%), 0 (0.0%)/2 (100.0%), 0 (0.0%)/2 (100.0%), and 16 (80.0%)/4 (20.0%), respectively. There were thirty-seven *RP1L1*, two *CRX*, two *GUCY2D*, and seventeen ND patients from whom mfERG data were available (Figure 3). The mfERG group 1/2/3 detected in the *RP1L1*, *CRX*, *GUCY2D*, and ND genotype groups was as follows: 3 (8.1%)/28 (75.7%)/6 (16.2%), 2 (100.0%)/0 (0.0%)/0 (0.0%), 0 (0.0%)/2 (100.0%)/0 (0.0%), and 4 (23.5%)/12 (70.6%)/1 (5.9%). SD-OCT data were available for forty-seven *RP1L1*, two *CRX*, two *GUCY2D*, and twenty-one ND patients with available SD-OCT data (Figure 3). The number of classical/non-classical SD-OCT findings in the *RP1L1*, *CRX*, *GUCY2D*, and ND genotype groups were 41 (87.2%)/6 (12.8%), 0 (0.0%)/2 (100.0%), 0 (0.0%)/2 (100.0%), and 1 (4.8%)/20 (95.2%), respectively.

### 3.7. Comparison Analyses among Genotype Groups

The following clinical parameters were compared among genotype groups; age, disease onset, disease duration, BCVA, VF classification, mfERG classification, and SD-OCT classification (Figure 2 and Figure 3). No statistically significant difference was found regarding the clinical parameters, although there was a trend of earlier onset and more severe BCVA in the *RP1L1* and *GUCY2D* genotype groups. The proportions of the VF pattern, mfERG group, and SD-OCT classification were compared among the genotype groups. A severe VF pattern with a central scotoma was frequently detected in the *GUCY2D*, ND, and *RP1L1* genotype groups. mfERG group 2 was frequently found in the *RP1L1*, *GUCY2D*, and ND genotype groups. Classical SD-OCT findings were detected in forty-one patients (41/47, 87.2%) of the *RP1L1* genotype group, while only one case with classical SD-OCT findings was detected in the ND genotype group (1/21, 4.8%).

### 3.8. Genotype R+ Data Set

For the *GUCY2D* genotype group, BCVA data of 58 previously reported cases in 29 families with the identical variant to those detected in this study were incorporated (Table 3 and Table S3) [16,40,48,49,50,51,52,53,54,55,56,57,58,59,60,61]. Three families with p.I916T and fifty-three families with p.R838H were included in the *GUCY2D* genotype R+ cohort. No previously reported cases with identical variants were detected in the *CRX* genotype group. The median age at onset/examination in the *GUCY2D* genotype R+ dataset was 7.5 (range, 1–55 years)/28 (range, 2.5–71). The median duration was 13 years (range, 0–32) and the medial logMAR BCVA in the right and left eye was 0.80 (range, 0.03–2.30) and 0.60 (range, 0.02–2.70), respectively. The phenotypic features of cone dystrophy (COD) and con-rod dystrophy (CORD) were documented in 37 and 20 patients (63.8% and 34.5%, respectively).

### 3.9. Kaplan-Meier Survival Analyses for BCVA

**Figure 4:**
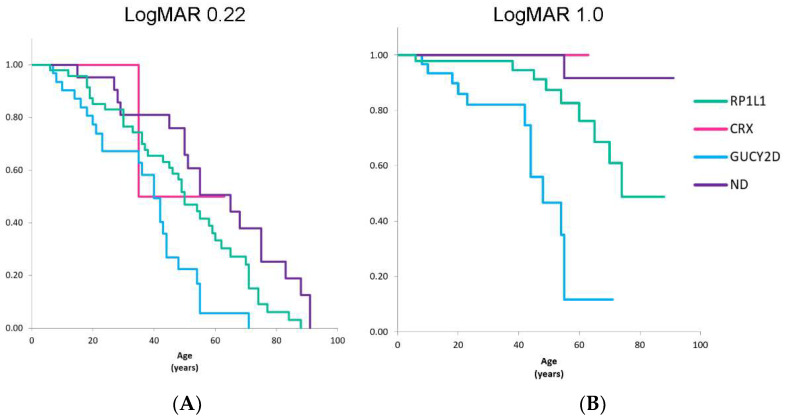
Survival curve analyses of BCVA for the genotype groups. Survival curves of BCVA for age were generated for the *RP1L1*, *CRX*, *GUCY2D*, and ND genotype groups in terms of two BCVA levels: (**A**) logMAR BCVA of 0.22 and (**B**) logMAR BCVA of 1.00. For the *GUCY2D* genotype group, the BCVA data of 58 previously reported cases from 29 families with identical variants detected in this study were incorporated. Approximately half of the patients in the *RP1L1* genotype group reached a BCVA level of 0.22 at age 49, while approximately half of the patients in the *GUCY2D* genotype group reached that level at age 36. Thus, there was a 13-year difference in VA reduction between the *RP1L1* and *GUCY2D* genotype groups. Approximately half of the patients in the *RP1L1* genotype group reached a VA level of 1.00 at age 74, while approximately half of the patients in the *GUCY2D* genotype group reached that level at age 44; and in the other genotype groups, most patients (>80%) did not reach 1.00. A statistically significant difference was revealed between OMDs in terms of survival curves of BCVA (*p* < 0.01). VA, visual acuity; ND, not detected; OMD, occult macular dystrophy; BCVA, best-corrected visual acuity; logMAR, logarithm of minimum angle of resolution. For the *GUCY2D* genotype group, logMAR BCVA data of fifty-eight previously reported cases in twenty-nine families with the identical variant to those detected in this study were incorporated and four of these cases presented normal fundus appearance.

Kaplan-Meier survival analysis was used to assess BCVA (logMAR 0.22 and logMAR 1.00). The BCVA data of forty-seven *RP1L1*, two *CRX*, fifty-eight *GUCY2D* R+, and twenty-one ND genotype patients were analysed (Figure 4). The diagram for logMAR 0.22 indicates that approximately half of the patients in the *RP1L1* genotype group reached a BCVA level of 0.22 at age 49, while approximately half of the patients in the *GUCY2D* group reached that level at age 36. Thus, there was a 13-year difference in VA reduction between the *RP1L1* and *GUCY2D* genotype groups. The diagram for logMAR 1.00 shows that approximately half of the patients in the *RP1L1* genotype group reached a VA level of 1.00 at age 74, while approximately half of the patients in the *GUCY2D* genotype group reached that level at age 44; and in the other genotype groups, most patients (>80%) did not reach 1.00.

## 4. Discussion

**Figure 5:**
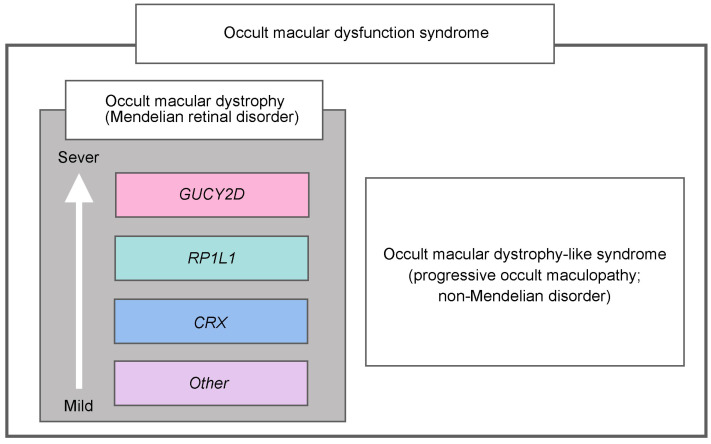
Clinical entity of occult macular dysfunction syndrome. The clinical entities of occult macular dysfunction syndrome have been established, including Mendelian hereditary disorders (*RP1L1*-OMD, *CRX*-OMD, *GUCY2D*-OMD, other hereditary OMD) and OMD-like non-Mendelian disorder (progressive occult maculopathy). OMD, occult macular dystrophy.

The clinical and genetic spectrum of OMDS, illustrating macular dysfunction with a normal fundus appearance, has been comprehensively outlined, identifying different severities and prognoses based on each genotype group. New clinical entities of OMDS have been established, including Mendelian hereditary disorders (*RP1L1*-OMD, *CRX*-OMD, *GUCY2D*-OMD, and other hereditary OMD) and OMD-like non-Mendelian disorder (progressive occult maculopathy) (Figure 5).

In the current study, 75% of patients with OMDS had a chief complaint of VA decline. We observed a trend of different severities of BCVA based on each genotype group, although it did not reach statistical significance, and a statistically different survival curve of BCVA was observed among the genotype groups. The most severe BCVA curve was observed in the *GUCY2D* genotype group, and there was a 30-year gap between the *GUCY2D* and *RP1L1* genotype groups in terms of social blindness (logMAR 1.00). In contrast, the *CRX* and ND genotype groups exhibited better VA curves. These differences in clinical severity were consistent with the distinct molecular mechanisms of *RP1L1*-retinopathy, *CRX*-retinopathy, and *GUCY2D*-retinopathy [63,64,65,66,67,68,69,70,71,72,73,74,75,76,77,78,79,80,81,82,83].

*GUCY2D*, denoted as guanylate cyclase 2D (OMIM: 600179), encodes one of the two retinal membrane guanylyl cyclase isozymes expressed on photoreceptors [62,84]. *GUCY2D*-retinopathy encompasses severe AR-Leber congenital amaurosis (AR-LCA), AR-RP, AD-CORD, AD-COD, and AD-MD [40,56,62]. Most patients with *GUCY2D*-AD-COD/CORD show progressive atrophic fundus abnormalities, which is consistent with the more severe and progressive phenotype of *GUCY2D*-OMD than that of *RP1L1*-OMD. Considering the AD-COD/CORD cases with retinal atrophy, patients with *GUCY2D*-OMD in this study could potentially develop visible macular atrophy with age. *CRX*, a cone–rod homeobox-containing gene (OMIM: 602225), encodes a homeodomain transcription factor crucial for the development and survival of photoreceptors [85,86]. *CRX*-retinopathy encompasses severe AR-LCA, AR-RP, AD-CORD, AD-COD, and AD-MD; AD-MD shows a mild phenotype [39,87,88,89,90,91,92,93,94,95,96,97,98,99,100,101]. The relatively mild phenotype of patients with *CRX*-OMD is consistent with the previous AD-*CRX* cases [87], although there are no reported *CRX* cases with the identical variant.

The clinical classification of the VF pattern, mfERG, and SD-OCT demonstrated different features based on each genotype group. A severe VF pattern with a central scotoma was frequently detected in the *GUCY2D*, ND, and *RP1L1* genotype groups. Homogeneous central dysfunction with preserved peripheral function (mfERG group 2) was frequently observed in patients with OMDS, except in those in the *CRX* genotype group. Other scotoma patterns and foveal functional preservation were observed in the *CRX* genotype group, which may be related to the bull’s eye changes. Classical SD-OCT findings were detected in most (>80%) patients in the *RP1L1* genotype group and these features were almost exclusively specific to *RP1L1*-OMD. Non-classical SD-OCT findings have been reported in mild cases with *RP1L1*-OMD [6]. However, in the current study, such morphological findings were demonstrated both in the severe and the mild genotype groups (*GUCY2D* and *CRX*, ND).

In this study, causative genes were not detected in 21 patients. Seven families (7/17, 41.2%) reported a family history of AD/AR (5/2), and unrevealed Mendelian hereditary disorders were included in this ND genotype group. However, the presence of non-hereditary disorders (e.g., occult maculopathy) cannot be excluded, given the elderly cases (e.g., 89-year-old female with AD inheritance in a family with three affected members across the two generations; and a sibship pair of a 91-year-old female and an 88-year-old male with AR inheritance in a consanguineous family). Variable pathologies presenting presumably AD, AR, and other inheritance patterns potentially underlying the ND genotype group can support a wide range of clinical parameters, including onset and BCVA. Interestingly, survival curve analyses suggested a mild prognosis. More detailed genetic analyses could reveal further causative genes, which would help to clarify the mechanisms of the ND genotype group in this study.

This study has several limitations. First, the sequencing methods applied, the selection of analysed genes, and the pathogenicity prediction protocols were rigorous but not absolute. Therefore, uncertainties may remain, and the results may not be completely exact. We did not analyse genes that were not registered in RetNet, possibly overlooking some genetic factors tied to OMDS pathologies. Whole-genome analysis could reveal more genetic irregularities in unresolved familial cases. Although the in silico analyses offer preliminary insights into potential genetic pathogenesis, these results are speculative and necessitate further verification. Second, this study cohort mainly comprises adult participants, with fewer pediatric or late-onset cases. Since some patients remain asymptomatic, our findings may not represent the entire disease spectrum. Rigorous clinical examinations including colour vision testing and genetic screening, especially for patients at risk of IZ disappearance, are crucial for early detection. Third, the intrafamilial variability in terms of the onset of the disease was observed in 16 families with multiple affected family members. This may be because of the molecular mechanisms of dominant negative/gain of function in AD disorders. However, a larger cohort would be valuable to elucidate the intrafamilial variation. Finally, this cross-sectional, retrospective study provides a snapshot of the genetic background of the East Asian population. Future longitudinal studies of global populations could better map the epidemiology, progression, and underlying mechanisms of OMDS.

## 5. Conclusions

This multicentre study, representing the largest cohort to date, significantly broadens our understanding of the phenotypic and genotypic spectra of patients with macular dystrophy who exhibit a normal fundus appearance. OMDS includes multiple Mendelian retinal disorders and beyond, with each presenting distinct pathologies that determine their specific severity and prognostic trajectory. These features enrich the accurate clinical and genetic diagnosis, which could inform patient monitoring and counselling, as well as the design of future therapeutic trials.

## Figures and Tables

**Table 1:** Demographic data and clinical classifications of 72 patients from 50 families with occult macula dysfunction syndrome by genotype.

		*RP1L1*	*CRX*	*GUCY2D*	Other OMD
Inheritance (number of families)	AD	23	1	1	5
	AR	0	0	0	2
	Sporadic	7	0	1	10
Age (years) ^†^		49 (6–88)	54 (40–68)	47.5 (40–55)	55 (15–91)
Age at onset (years) ^†^		30 (2–71)	49.5 (31–68)	26.5 (13–40)	45 (6–49)
Disease duration (years) ^†^		11 (0–63)	4.5 (0–9)	21 (0–42)	8 (0–51)
BCVA (logMAR) ^†^	Right eye	0.52 (−0.08–1.52)	0.07 (−0.08–0.22)	0.91 (0.82–1)	0.4 (0.18–1.4)
	Light eye	0.52 (−0.08–1.7)	0.16 (−0.08–0.4)	0.96 (0.82–1.1)	0.4 (0.18–1.4)
Visual field pattern ^††^	Pattern 1	32	0	2	16
	Pattern 2	11	2	0	4
mfERG group ^§^	Group 1	3	2	0	4
	Group 2	28	0	2	12
	Group 3	6	0	0	1
SD-OCT classification ^§§^	Classical	41	0	0	1
	Non-classical	6	2	2	20

AD, autosomal dominant; AR, autosomal recessive; BCVA, best-corrected visual acuity; logMAR, logarithm of minimum angle of resolution; mfERG, multifocal electroretinogram; RE, right eye; SD-OCT, spectral-domain optic coherence tomography. ^†^ The median value and range of clinical parameters for each genotype group are provided. ^††^ Patients were classified into two patterns based on the results of VF testing using standard automated perimetry: Pattern 1, central scotoma; Pattern 2, other scotomas (e.g., paracentral scotoma), or no scotoma, mainly according to a previous publication. ^§^ Patients were classified into three objective functional groups based on mfERG findings: Group 1, paracentral dysfunction with relatively preserved central/peripheral function; Group 2, homogeneous central dysfunction with preserved peripheral function; and Group 3, widespread dysfunction over the recorded area, according to a previous publication. ^§§^ Classical Spectral-domain Optical Coherence Tomography (SD-OCT) findings were marked as a blurred ellipsoid zone (EZ) and an absence of an interdigitation zone (IZ) at the macula. Conversely, non-classical findings present alterations incongruent with the typical phenotype, including a locally absent IZ while retaining a relatively preserved EZ, a focal absence of both the EZ and IZ, or atrophic transformations within the outer retinal layers.

**Table 2:** Summary of causative genes and variants in 33 families with occult macular dystrophy.

Gene	Nucleotide Change, Amino Acid Change	State	Family Number	Reference
*RP1L1*	c.133C>T, p.Arg45Trp	Het	16	Akahori et al. (2010) [33], Tsunoda et al. (2012) [25], Fujinami et al. (2019) [6], Wang et al. (2020) [15]
*RP1L1*	c.3581C>T, p.Thr1194Met/c.3587C>T, p.Thr1196Ile	Het	1	Fujinami et al. (2016) [11]
*RP1L1*	c.3593C>T, p.Ser1198Phe	Het	1	Fujinami et al. (2019) [6]
*RP1L1*	c.3595T>C, p.Ser1199Pro	Het	1	Takahashi H et al. (2014) [31], Fujinami et al. (2019) [6]
*RP1L1*	c.3596C>G, p.Ser1199Cys	Het	7	Kabuto et al. (2012) [32], Fujinami et al. (2019) [6]
*RP1L1*	c.3599G>A, p.Gly1200Asp	Het	1	Fujinami et al. (2016) [11]
*RP1L1*	c.3599G>C, p.Gly1200Ala	Het	1	Fujinami et al. (2019) [6]
*RP1L1*	c.3602T>G, p.Val1201Gly	Het	1	Fujinami et al. (2016) [11]
*RP1L1*	c.3602T>C, p.Val1201Ala	Het	1	This study
*CRX*	c.128G>A, p.Arg43His	Het	1	Fujinami-Yokokawa et al. (2020) [39]
*GUCY2D*	c.2747T>C, p.Ile916Thr	Het	1	de Castro-Miró et al. (2014) [49], Liu et al. (2020) [40]
*GUCY2D*	c.2513G>A, p.Arg838His	Het	1	Payne et al. (2001) [48], Liu et al. (2020) [40]

OMDS, occult macular dysfunction syndrome; Het, heterozygous. Reference: NM_178857.5, ENST00000382483.3, GRCh37; NM_000554.6 ENST00000221996.12,GRCh37,NM_000180.4, ENST00000254854.5GRCh37. Reference numbers described in the text are provided for previous publications.

**Table 3:** Previous reports of *CRX* and *GUCY2D* variants identified in this study.

Gene	Variant	Previous Report	Inheritance	Phenotype (Family Number)	Presence with Normal Fundus (Subject Number)
*CRX*	c.128G>A, p.Arg43His	Fujinami-Yokokawa et al. (2020) [59]	AD	OMD (1)	Yes (2)
*GUCY2D*	c.2747T>C, p.Ile916Thr	de Castro-Miró et al. (2014) [49]	AD	COD (1)	NA
		Liu et al. (2020) [40]	AD	OMD (1)	Yes (1)
		Rodilla C et al. (2023) [56]	AD	CORD (1)	NA
	c.2513G>A, p.Arg838His	Payne et al. (2001) [48]	AD	CORD (1)	No(1)
		Ito et al. (2004) [50]	AD	COD (1)	Yes (1), no (1)
		Weigell-Weber et al. (2000) [52]	AD	CORD (1)	NA
		Lazar et al. (2015) [60]	AD	COD (2), CORD (1)	No (2)
		Kim et al. (2019) [16]	AD	COD (1)	NA
		Sharon et al. (2019) [62]	AD	COD (1)	NA
		Udar et al. (2003) [61]	AD	CORD (1)	NA
		Kitiratschky et al. (2008) [54]	AD	COD (3)	Yes (1), no (6)
		Xiao et al. (2011) [58]	AD	COD (1)	No (8)
		Mukherjee et al. (2014) [55]	AD (de novo)	COD (1)	No (3)
		Zobor et al. (2014) [59]	AD	COD/CORD (1)	Yes (1), no (2)
		Jiang et al. (2015) [53]	AD	COD (4), CORD (1)	No (6)
		Sun et al. (2020) [57]	AD	CORD (1)	No (2)
		Rodilla C et al. (2023) [56]	AD	CORD (8)	NA

AD, autosomal dominant; OMD, occult macular dystrophy; OMDS, occult macular dysfunction syndrome; COD, cone dystrophy; CORD, cone rod dystrophy; MD, macular dystrophy; NA, not available. Reference numbers described in the text are provided for previous publications.

## Data Availability

The data presented in this study are available on request from the corresponding author. The data are not publicly available due to ethical rules and regulations.

## Supplementary Materials

The following supporting information can be downloaded at: https://www.mdpi.com/article/10.3390/genes14101869/s1, Table S1: Detailed clinical and genetic findings of 72 patients from 50 families with occult macular dysfunction syndrome; Table S2: In silico analysis of the detected variants in this study; Table S3: Detailed clinical and genetic information of previously reported cases harbouring the *CRX* and *GUCY2D* variants detected in this study.
